# Amphetamine-Decreased Progesterone and Estradiol Release in Rat Granulosa Cells: The Regulatory Role of cAMP- and Ca^2+^-Mediated Signaling Pathways

**DOI:** 10.3390/biomedicines9050493

**Published:** 2021-04-29

**Authors:** Chung-Yu Chen, Chien-Rung Chen, Chiao-Nan Chen, Paulus S. Wang, Toby Mündel, Yi-Hung Liao, Shiow-Chwen Tsai

**Affiliations:** 1 Department of Exercise and Health Sciences, University of Taipei, Taipei City 111, Taiwan; fish0510@gmail.com; 2 Department of Nursing, Cardinal Tien Junior College of Healthcare and Management, New Taipei City 231, Taiwan; jjchen@ctcn.edu.tw; 3 Department of Physical Therapy and Assistive Technology, National Yang Ming Chiao Tung University, Taipei City 112, Taiwan; chiaonanchen@gmail.com; 4 Department of Physiology, National Yang Ming Chiao Tung University, Taipei City 112, Taiwan; pswang3879@gmail.com; 5 Medical Center of Aging Research, China Medical University Hospital, Taichung City 404, Taiwan; 6 Department of Biotechnology, College of Health Science, Asia University, Taichung City 413, Taiwan; 7 Department of Medical Research, Taipei Veterans General Hospital, Taipei City 112, Taiwan; 8 School of Sport, Exercise and Nutrition, Massey University, Palmerston North 4442, New Zealand; T.Mundel@massey.ac.nz; 9 Department of Exercise and Health Science, National Taipei University of Nursing and Health Sciences, Taipei City 112, Taiwan; 10 Institute of Sports Sciences, University of Taipei, Taipei City 112, Taiwan

**Keywords:** reproductive hormones, follicle-stimulating hormone (FSH) administration, steroidogenic enzymes, protein kinase A (PKA), L-type calcium channel

## Abstract

The purpose of this study is to evaluate the amphetamine effects on progesterone and estradiol production in rat granulosa cells and the underlying cellular regulatory mechanisms. Freshly dispersed rat granulosa cells were cultured with various test drugs in the presence of amphetamine, and the estradiol/progesterone production and the cytosolic cAMP level were measured. Additionally, the cytosolic-free Ca^2+^ concentrations ([Ca^2+^]i) were measured to examine the role of Ca^2+^ influx in the presence of amphetamine. Amphetamine in vitro inhibited both basal and porcine follicle-stimulating hormone-stimulated estradiol/progesterone release, and amphetamine significantly decreased steroidogenic enzyme activities. Adding 8-Bromo-cAMP did not recover the inhibitory effects of amphetamine on progesterone and estradiol release. H89 significantly decreased progesterone and estradiol basal release but failed to enhance a further amphetamine inhibitory effect. Amphetamine was capable of further suppressing the release of estradiol release under the presence of nifedipine. Pretreatment with the amphetamine for 2 h decreased the basal [Ca^2+^]i and prostaglandin F2α-stimulated increase of [Ca^2+^]i. Amphetamine inhibits progesterone and estradiol secretion in rat granulosa cells through a mechanism involving decreased PKA-downstream steroidogenic enzyme activity and L-type Ca^2+^ channels. Our current findings show that it is necessary to study the possibility of amphetamine perturbing reproduction in females.

## 1. Introduction

Amphetamine, an indirect dopamine agonist, was first discovered 100 years ago. Since then, numerous studies have confirmed that amphetamine influences the central and peripheral nervous system by acting on the activities of monoamine reuptake transporters. The functional responses altered by amphetamine include reduced reaction time [1], anti-fatigue [2] and impaired cognition [3]. An acute overdose of amphetamine causes impairment of executive brain function and leads to severe drug addiction [4], and chronic intake of amphetamine can be associated with grave and even fatal side-effects [5]. Furthermore, Huybrechts et al., reported that amphetamine exposure in pregnancy will increase the risk of congenital malformations compared with no exposure to stimulants [6]. There is poor obstetric history in women addicted to amphetamine including a high incidence of previous abortion, preeclampsia, infection and antepartum hemorrhage [7]. In endometrial tissue, progesterone levels are 200 times higher in fertile women than in those with habitual miscarriages; however, in women with recurrent miscarriages, both progesterone (PgR) and estradiol (ER) receptors are at their lowest levels in the cytoplasmic and nuclear regions [8]. Because the plasma and endometrium tissue levels of progesterone play a key role in the biosynthesis of ER and PgR [8], these above findings suggest that the abuse of amphetamine possibly results in several abnormal female endocrinological responses and perturbations in reproductive function through the dysregulation of female sex hormones.

Amphetamine’s impacts on the endocrinological system have not been thoroughly investigated, despite several investigations having examined the impacts of amphetamine on the male reproductive system [9,10]. Our previous results demonstrated that amphetamine inhibits both basal and human chorionic gonadotropin (hCG)-stimulated testosterone release in vivo [9] and in vitro [9,10] via increased adenosine 3′:5′-cyclic monophosphate (cAMP) production, decreased Ca^2+^ influx through L-type calcium channel and decreased 3β-hydroxysteroid dehydrogenase (3β-HSD), 17α-hydroxylase/C17-20 lyase (P450c17) and 17β-hydroxysteroid dehydrogenase (17β-HSD) activities [10]. Moreover, previous reports showed that amphetamine has multiple effects stimulating dopamine release [11] and influences other hormones’ release [12,13,14]. Methamphetamine, an analog of amphetamine, impairs testes function through morphology damage [15], apoptosis induction [16,17], decreased spermatogenesis [18] and testosterone secretion [15]. Furthermore, amphetamine inhibits lordosis in ovariectomized rats treated with estrogen [19]. According to the similarity in sex hormone production between genders, this implies that amphetamine could impair female reproductive physiological patterns by perturbing the hormonal system. However, amphetamine’s effects on female sex hormone secretion, such as progesterone released from granulosa cells, are still poorly understood, although the above previous reports revealed a clear negative impact of amphetamine on male reproductive hormonal regulation.

Female sex hormone production is complex and regulated by interactions between granulosa cells and theca cells. Progesterone is the main secretory product of granulosa cells and diffuses into theca cells to serve as a substrate for androgen biosynthesis [20,21,22]. Thereafter, theca cells subsequently release androgens for granulosa cells to convert androgens into estrogens. The granulosa cell, therefore, plays a primary role in initiating progesterone and estrogen production in response to follicle-stimulating hormone (FSH) stimulation [21]. FSH-induced progesterone release is dually regulated through two distinct intracellular signaling systems, including adenyl cyclase/cAMP- and L-type calcium channel-mediated pathways. FSH increases progesterone [20,21,23,24,25] and estradiol production [20,24,26], which is regulated via the cAMP-related signaling pathway [23,24,26]. It further activates P450scc (cytochrome P450 side-chain cleavage), 3β-HSD or P450arom in granulosa cells [25,27,28,29]. On the other hand, FSH also activates the L-type calcium channel system, thereby increasing [Ca^2+^]i and calcium-mediated progesterone biosynthesis [30].

This investigation determines whether amphetamine perturbs progesterone and estradiol production in response to FSH stimulation in rat granulosa cells. We further investigated the underlying cellular mechanisms for amphetamine’s actions on these sex hormone production pathways, including (i) cAMP-regulatory cascade, (ii) the enzyme activities controlling steroidogenesis (i.e., P450scc, 3β-HSD, 17β-HSD and P450arom) and (iii) L-type calcium channel activation.

## 2. Materials and Methods

### 2.1. Reagents

Chemicals and reagents including pregnant mare serum gonadotropin (PMSG), Dulbecco’s modified Eagle medium (DMEM)/F12, fatty acid-free bovine serum albumin (BSA), N-2-hydroxyethlypiperazine-N’-2-ethanesulphonic acid (HEPES), penicillin-G, streptomycin sulfate, insulin, medium-199 (M199), L-glutamine, amphetamine, 3-isobutyl-1-methylxanthine (IBMX), 8-bromo-cAMP (8-Br-cAMP), nifedipine, 25-OH-cholesterol, pregnenolone, androstenedione, testosterone and prostaglandin F2α (PGF2α) were purchased from Sigma Chemical Co. (St. Louis, MO, USA). Fura-2/AM and H89 dihydrochloride (H89) were purchased from Calbiochem-Novabiochem Corp. (San Diego, CA, USA). [^3^H]-Pregnenolone, [^3^H]-androstenedione, [^3^H]-progesterone and [^3^H]-estradiol were obtained from Amersham International plc. (Buckinghamshire, UK). Fetal calf serum was obtained from UBI (Kibbutz Beit Haemek, Israel). Porcine follicle-stimulating hormone (pFSH) was provided by the National Hormone and Pituitary Program of the National Institute of Child Health and Human Development and the U.S. Department of Agriculture, USA. Thin-layer chromatography (TLC) plates (0.25 mm thick silica gel G sheets precoated with fluorescent indicator, 20 × 20 cm) were purchased from Macherey-Nagel (Duren, Germany). Cell culture plasticware was obtained from Falcon Labware (Lincoln Park, NJ, USA).

### 2.2. Granulosa Cell Isolation and Culture

Immature female Sprague Dawley rats were purchased from the National Laboratory Animal Center, Taipei, Taiwan. All animals were housed in the animal center of Shin Kong Wu Ho-Su Memorial Hospital under a temperature-controlled environment (22 ± 1 °C) with 14 h of artificial illumination daily (06:00–20:00 h) and were given food and water ad libitum. All animal experiments performed in this study were approved by the Institutional Animal Care and Use Committee of the Shin Kong Wu Ho-Su Memorial Hospital (IACUC approval no. 051228001). Granulosa cell preparation was modified from the method described by Too [22]. Immature female rats at 22–25 days of age were subcutaneously injected with PMSG (15 IU/rat). The rats were sacrificed by cervical dislocation at 48-h after PMSG injection. Ovaries were excised and transferred into sterile DMDM/F12 (1:1) medium, containing 0.1% BSA, 20 mM HEPES, 100 U/mL penicillin-G and 50 μg/mL streptomycin sulfate. After trimming the fat and connective tissues, the surface of large and medium-sized follicles was punctured with a 26-gauge needle to release granulosa cells. The procedure was carefully operated under a microscope to avoid possible contamination with other interstitial cells. The harvested cells were pelleted and resuspended in a growth medium (DMEM/F12 containing 10% fetal calf serum, 2 μg/mL insulin, 100 IU/mL penicillin and 100 μg/mL streptomycin sulfate). Cell viability was greater than 90% as determined using a hemocytometer and trypan blue method. Rat granulosa cells were plated in 24-well plates at approximately 1 × 10^5^ cells per well and incubated at 37 °C with 5% CO_2_-95% air for two days. Morphologically, the cultured granulosa cells maintained a characteristic round (or polygonal) shape throughout our culture conditions [25,31]. Purified granulosa cells from large and medium follicles displayed steroidogenic criteria [20]: (i) the granulosa cells did not synthesize estradiol unless aromatized androgens (i.e., androstenedione and testosterone) were added, and (ii) FSH significantly stimulated progesterone production in granulosa cells. When conducting the cell culture and reagent incubation experiments, we performed at least four independent experiments as in previous literature [22,25,31]. Total cell proteins were determined using the method of Lowry et al. [32]. The incubation concentrations of amphetamine treatment were selected in accordance with a previous clinical dose-response study by Angrist et al., (1987), of which the plasma amphetamine levels ranged between approximately ~2.2–5.2 × 10^−7^ M and peaked at 2–3 h after an oral administration (0.25–0.5 mg/kg) [33]. Therefore, we tested the cellular responses under the conditions with amphetamine at 10^−8^–10^−6^ M for 2 h incubation to better mimic the physiological environment of amphetamine administration.

### 2.3. Amphetamine Effects on Progesterone, Estradiol and cAMP Production in Granulosa Cells

The granulosa cells were washed twice using a BSA-M199 medium (M199 without phenol red, 0.3% BSA, 25 mM HEPES, 4 mM L-glutamine) and then incubated with 500 μL aliquots of serum-free BSA-M199 medium. Amphetamine (10^−8^–10^−6^ M), pFSH (10 ng/mL) or pFSH plus amphetamine in 500 μL fresh medium in the absence or presence of IBMX was added to the wells. To evaluate estradiol production, androstenedione was added to a final concentration of 10^−8^ M. After incubation for 2 h at 37 °C in 5% CO_2_ and 95% air, media were collected and cleared by centrifugation. The supernatants were stored at −20°C until analyzed for progesterone [25,31] and estradiol [34] using radioimmunoassay (RIA). For the analysis of cAMP production in response to amphetamine, cells were primed for 30 min and then incubated for 2 h with 500 μL medium containing 0.5 mM IBMX. IBMX, a competitive non-selective phosphodiesterase inhibitor, was added in the incubation medium to sustain the inducible cAMP levels [9,10]. At the end of incubation, the intracellular cAMP was extracted using 65% ethanol as previously described [25]. The supernatants were lyophilized in a vacuum concentrator (Speed Vac, Savant, Holbrook, NY, USA) and stored at −20°C until analyzed for cAMP using RIA [10,35].

### 2.4. Amphetamine Effects on cAMP- and Ca^2+^-Induced Progesterone and Estradiol Production

To further evaluate the role of intracellular cAMP and Ca^2+^ in progesterone and estradiol release regulation by amphetamine, 8-Br-cAMP (a membrane-permeable analog of cAMP to mimic increased intracellular cAMP, 10^−4^ or 10^−3^ M) [24], H89 (an inhibitor of protein kinase A catalytic subunit, 5 × 10^−9^ or 5 × 10^−8^ M) and nifedipine (L-type calcium channel blocker, 10^−8^–10^−6^ M) [10] were applied. After priming for 30 min, a fresh BSA-M199 medium (500 μL) containing amphetamine (10^−8^/10^−6^ M) was added to the wells to determine the amphetamine effect influenced by intracellular cAMP and Ca^2+^. To evaluate estradiol production, androstenedione was added to a final concentration of 10^−8^ M. After incubation at 37 °C with 5% CO_2_ and 95% air for 2 h, media were then collected, centrifuged and stored at −20 °C until analyzed for progesterone and estradiol using RIA.

### 2.5. Amphetamine Effect on Steroidogenic Enzyme Activities

To ascertain the activities of steroidogenic enzymes separately, precursors including 25-OH-cholesterol (a substrate of P450scc that readily passes through the cell and mitochondrial membrane), pregnenolone (a substrate of 3β-HSD), androstenedione (a substrate of 17β-HSD) or testosterone (acts as a substrate of P450arom) were added. Granulosa cells were incubated with a fresh BSA-Med 199 medium containing the precursors (0, 10^−7^–10^−5^ M) [35] including 25-OH-cholesterol, pregnenolone in the absence or presence of amphetamine (0, 10^−8^–10^−6^ M). Two hours later, the medium was collected and analyzed for progesterone by RIA. For measurement of estradiol, either androstenedione or testosterone (0, 10^−7^–10^−5^ M) was added into the medium.

Steroidogenic enzyme activities were determined using TLC as previously described [10,25]. Granulosa cells were incubated with [^3^H]-pregnenolone or [^3^H]-androstenedione (10,000 cpm, 0.2 pmol) in the absence or presence of amphetamine (10^−10^–10^−6^ M) for 2 h. The medium was extracted by agitation in 1 mL diethyl ether and then frozen in an acetone mixture using dry ice. The diethyl ether layers were collected, dried and reconstituted in 100 μL absolute ethanol containing 5 μg of each of the unlabeled carriers, including pregnenolone, progesterone and 17α-hydroxyprogesterone. Aliquots (50 μL) were applied to a TLC plate (Macherey-Nagel, Duren, Germany) and separated using a carbon tetrachloride and acetone mixture (4:1, vol/vol ratio). The sheets were dried and the steroid-containing spot locations were indicated under UV light. The migration rate (R*f*) values were 0.55 for pregnenolone, 0.71 for progesterone and 0.50 for 17α-hydroxyprogesterone [10,35]. To distinguish androstenedione from estradiol in the presence of [^3^H]-androstenedione, the added carriers included androstenedione, testosterone and estradiol. The TLC sheets were developed in an n-heptane and acetone mixture (4:1, vol/vol ratio). The Rƒ value was 0.4 for androstenedione, 0.22 for estradiol and 0.11 for testosterone. The spots were cut off and transferred into vials containing 1 mL of liquid scintillation fluid (Ready Safe, Beckman, Fullerton, CA, USA) for the later radioactivity counting using an automatic beta counter (Wallac 1409, Pharmacia, Turku, Finland).

### 2.6. Intracellular Ca^2+^ Concentration Measurement

The cells were resuspended in a concentration of 1 × 10^7^/mL in the growth medium. Aliquots (1 mL) of the cells were loaded with Fura-2/AM (5 μL) for 30 min at 37 °C, and then centrifuged at 1000 r.p.m. for 10 min before being rinsed twice with loading buffer (150 mM NaCl, 5 mM KCl, 2 mM CaCl_2_, 1 mM MgCl_2_, 5 mM glucose and 10 mM HEPES at pH 7.4) to remove the excess Fura-2/AM. The cells were resuspended with loading buffer, in a final concentration of 1 × 10^6^/mL at room temperature, and kept in darkness until further use. [Ca^2+^]i was measured using the Fura-2-Ca^2+^ method, in which the fluorescence of Ca^2+^ was determined by SPEX (Model CM1T111, Industries, Inc., Edison, NJ, USA) according to the method originally described by Grynkiewicz et al. [36]. Briefly, cells were excited at 340 and 380 nm, respectively, and emission was measured at 505 mm. Rodway et al. demonstrated that the [Ca^2+^]i of rat granulosa cells was responsive to PGF2α at concentrations ranging from 10^−7^ to 10^−4^ M [37]. In the present study, PGF2α at final concentrations of 100 nM and 500 nM were mixed with the cells to stimulate Ca^2+^ mobilization. The amphetamine effect was investigated by preincubating the cells with 10–6 M amphetamine for 2 h before the addition of PGF2α, and the value of [Ca^2+^]i was recorded for 10 min. In these experiments, cells were lysed with 0.02% (wt/vol) digitonin, and 500 mM EGTA was added to obtain fluorescence values of fura-2 at both wavelengths (340 and 380 nm) under the condition of calcium saturation or depletion. The [Ca^2+^]i levels were calculated according to Grynkiewicz et al. [36].

### 2.7. RIAs of Progesterone, Estradiol, and cAMP

The progesterone level in the medium was determined using RIA as described previously [25]. With anti-progesterone serum no. W5, the progesterone RIA sensitivity was 15.4 pg/mL. The intra-and interassay coefficients of variation (CV) were 4.8% (*n* = 5) and 9.5% (*n* = 4), respectively. The estradiol concentration in the medium was determined by RIA as previously described [31]. With anti-estradiol serum no. W1, the estradiol RIA sensitivity was 3.5 pg/mL. The intra-and interassay CVs were 6.0% (*n* = 5) and 5.9% (*n* = 5), respectively. The cAMP concentration was determined by RIA as described elsewhere [9,10,34]. With anti-cAMP serum no. CV-27 pool, the cAMP RIA sensitivity was 10 fmol/mL. The intra-and interassay CVs were 6.9% (*n* = 5) and 11.9% (*n* = 5), respectively.

### 2.8. Statistical Analysis

All data were expressed as mean ± SEM. Treatment means were tested for homogeneity using the analysis of variance (ANOVA), and the differences between the specific means were tested for significance using Duncan’s multiple range test. The level of significance chosen was *p* < 0.05.

## 3. Results

### 3.1. Amphetamine Effects on Progesterone, Estradiol and cAMP Production in Granulosa Cells

**Figure 1:**
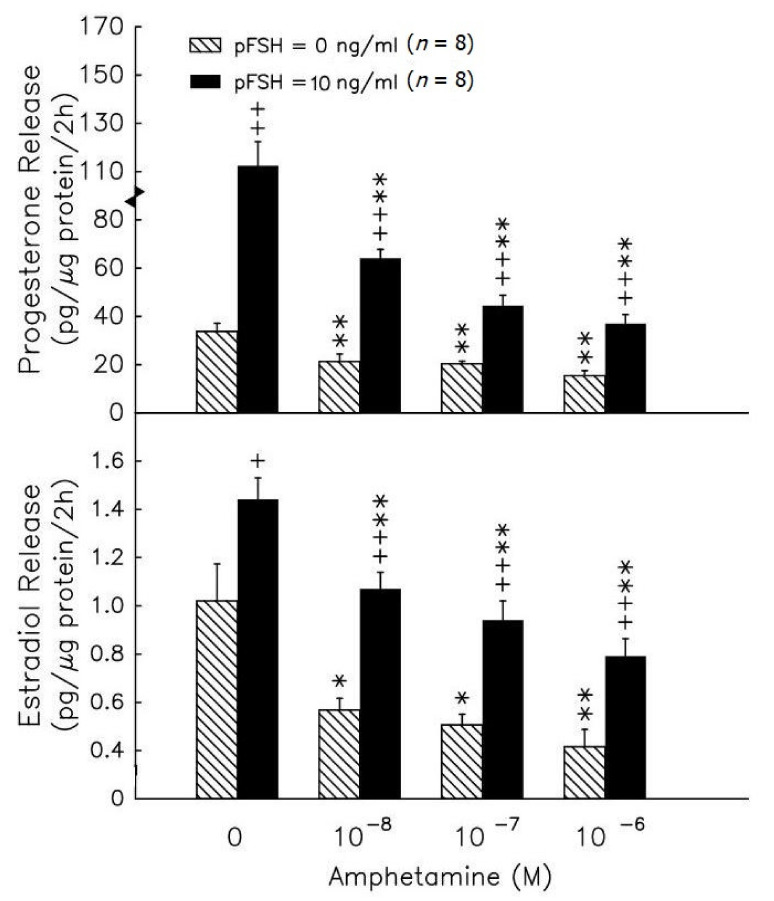
The in vitro effects of amphetamine on the release of progesterone (upper panel) and estradiol (lower panel) in rat granulosa cells. Granulosa cells were incubated with different doses of amphetamine in the presence (solid columns) or absence (hatched columns) of pFSH (10 ng/mL). To evaluate estradiol production, androstenedione was added to a final concentration of 10^−8^ M. After 2h, media were collected and stored at −20 °C until analyzed for progesterone and estradiol by RIA. * *p* < 0.05, ** *p* < 0.01 compared with amphetamine at 0 M, respectively. ^+^ *p* < 0.05, ^++^ *p* < 0.01 compared with non-pFSH-treated group, respectively. Each column represents mean ± s.e.m.

During the 2h incubation, amphetamine in the range of 10^−8^–10^−6^ M caused a dose-dependent inhibition of progesterone release by granulosa cells (*p* < 0.01, Figure 1, upper panel). In the presence of 10^−8^ M androstenedione, amphetamine in the range of 10^−8^–10^−^6 M inhibited estradiol release by granulosa cells in a dose-dependent manner (*p* < 0.05 or *p* < 0.01, Figure 1, lower panel). pFSH (10 ng/mL) stimulated both progesterone and estradiol secretion after the 2h treatment (*p* < 0.05 or *p* < 0.01). The combination of pFSH with amphetamine (10^−8^–10^−6^ M) significantly inhibited the pFSH-stimulated release of progesterone and estradiol (*p* < 0.01).

**Figure 2:**
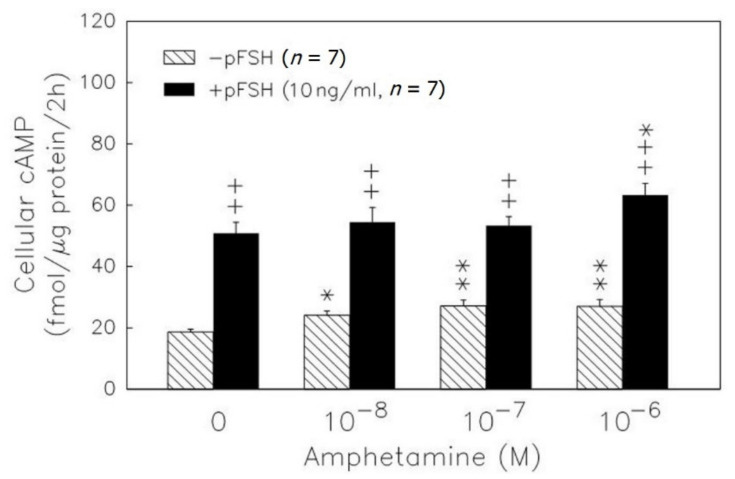
Dose-dependent effect of amphetamine on the accumulation of cAMP in rat granulosa cells. Granulosa cells were primed with 0.5 mM IBMX and then incubated with amphetamine combined with (solid column) or without (hatched column) pFSH. At the end of incubation, the intracellular cAMP was extracted and stored at −20 °C until analyzed by RIA. * *p* < 0.05, ** *p* < 0.01 compared with amphetamine at 0 M, respectively. ^++^ *p* < 0.01 compared with the non-pFSH-treated group. Each column represents mean ± s.e.m.

We further checked the cAMP intracellular level after treatments. pFSH administration significantly (*p* < 0.01) increased the cAMP accumulation in granulosa cells (Figure 2). Amphetamine ranging from 10^−8^ to 10^−6^ M increased the cAMP content (*p* < 0.05 or *p* < 0.01). Moreover, amphetamine at the dose of 10^−6^ M enhanced the pFSH-stimulated cyclic AMP accumulation (*p* < 0.05) in granulosa cells.

**Figure 3:**
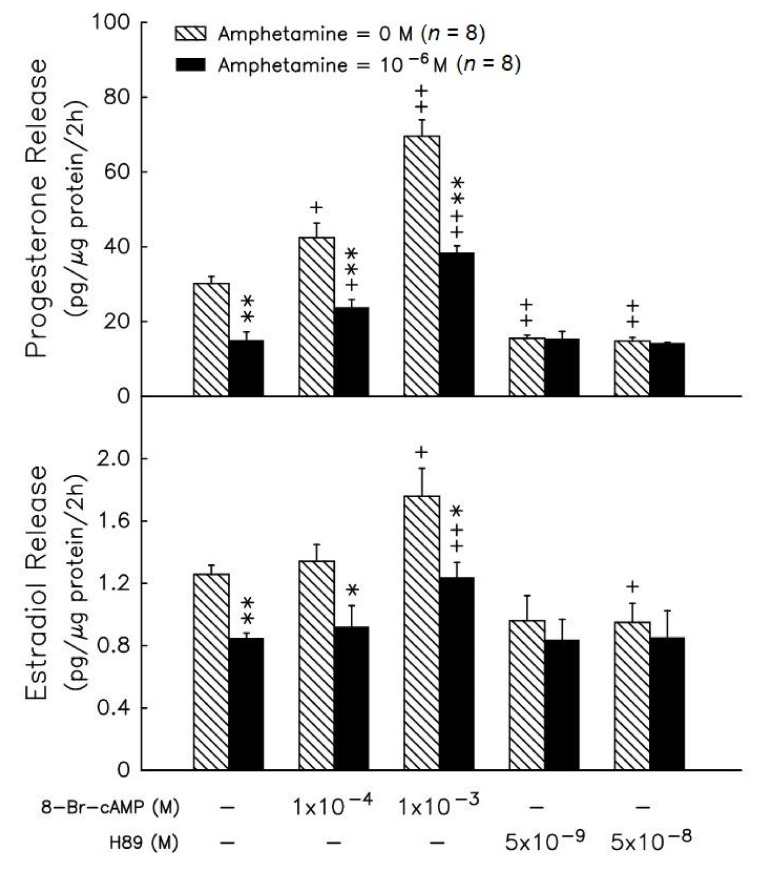
Effects of amphetamine on the release of progesterone (upper panel) and estradiol (lower panel) in rat granulosa cells in the presence of 8-Br-cAMP, or H89. * *p* *<* 0.05, ** *p* *<* 0.01 compared with amphetamine at 0 M, respectively. ^+^ *p* < 0.05, ^++^ *p* < 0.01 compared with corresponding control group, respectively. Each column represents mean ± s.e.m.

8-Br-cAMP at 10^−3^ M stimulated progesterone (*p* < 0.01) and estradiol release (*p* < 0.05) (Figure 3, upper panel), and progesterone and estradiol production inhibition by amphetamine was not recovered in granulosa cells (Figure 3). Administration of 5 × 10^−8^ M H89 (an inhibitor of protein kinase A) resulted in a decrease in the release of progesterone and estradiol (*p* < 0.05 or *p* < 0.01, Figure 3). The addition of H89 did not yield further suppressive effects of amphetamine on the release of estradiol and progesterone.

### 3.2. Amphetamine Effect on P450scc, 3β-HSD, 17βHSD, and P450 Arom Activities

**Figure 4:**
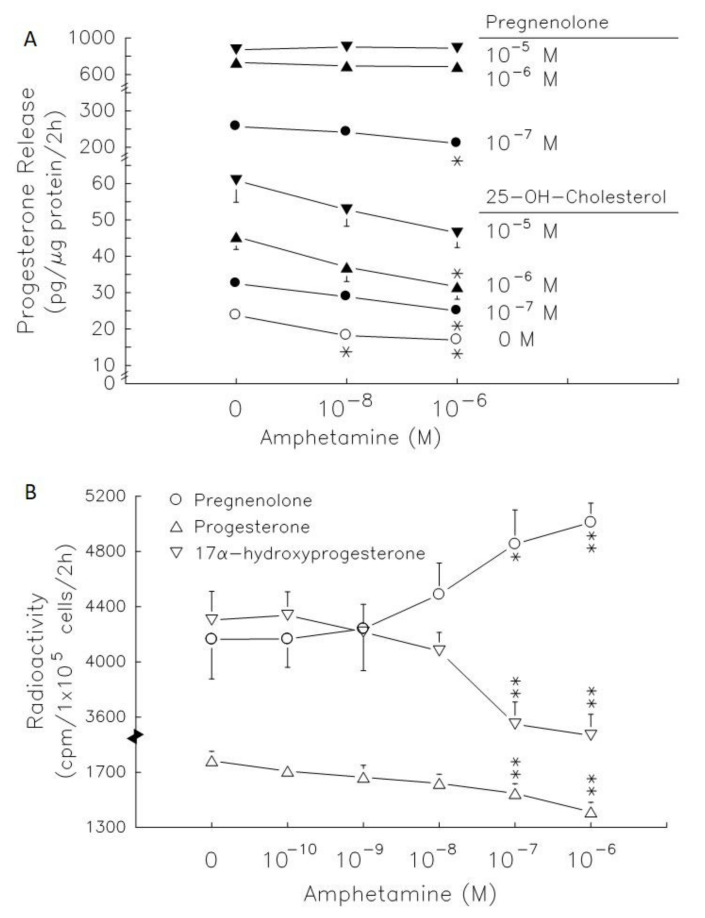
Effects of amphetamine on the activity of 3β-HSD in rat granulosa cells. (**A**) The release of progesterone following the presence of 25-OH-cholesterol or pregnenolone at different doses (○, 0 M; ●, 10^−7^ M; ▲, 10^−6^ M; ▼, 10^−5^ M). (**B**) Rat granulosa cells were incubated with [^3^H]-pregnenolone (10,000 cpm) and different doses of amphetamine at 37 °C for 2 h. The medium was extracted by ether, dried, and then reconstituted in ethanol before analysis by thin-layer chromatography (TLC). The radioactivities of [^3^H]-pregnenolone (○), [^3^H]-progesterone (△), and [^3^H]-17α-hydroxyprogesterone (▽) were measured. * *p* < 0.05, ** *p* < 0.01 compared with amphetamine at 0 M, respectively. Each symbol represents mean ± s.e.m.

Administration of 25-OH-cholesterol (Figure 4A) at doses ranging from 10^−^^7^–10^−^^5^ M increased progesterone release by 1.4-, 1.9- and 2.5-fold (*p* < 0.05 or *p* < 0.01). Amphetamine at 10^−^^6^ M resulted in a decrease in progesterone release of 32% in the absence of precursors, and of 23% and 30% in the presence of 10^−^^7^ M and 10^−^^6^ M 25-OH-cholesterol (*p* < 0.05, Figure 4A), respectively. Administration of pregnenolone (Figure 4, lower panel) at doses ranging from 10^−^^7^–10^−^^5^ M increased progesterone release by 11-, 31- and 37-fold (*p* < 0.01) and amphetamine at 10^−^^6^ M resulted in a decrease in progesterone release by 14% in the presence of 10^−^^7^ M pregnenolone (*p* < 0.05, Figure 4). The higher dose of 25-OH-cholesterol (10^−^^5^ M) and pregnenolone (10^−^^6^ and 10^−^^5^ M) reversed the inhibitory effect of amphetamine on progesterone release (Figure 4A).

The radiolabeled precursors could act as a tracer to help understand the synthesis of resultants. Therefore, incubation with [^3^H]-pregnenolone for 2 h, [^3^H]-progesterone, [^3^H]-17α-hydroxyprogesterone and [^3^H]-androstenedione (less than 100 cpm, data not shown) could be produced in granulosa cells (Figure 4). Meanwhile, [^3^H]-estradiol and [^3^H]-testosterone were produced after granulosa cell incubation with [^3^H]-androstenedione for 2 h (Figure 4). Amphetamine dose-dependently increased [^3^H]-pregnenolone accumulation, but decreased [^3^H]-progesterone and [^3^H]-17α-hydroxyprogesterone production (Figure 4B). The accumulation of [^3^H]-pregnenolone increased by 1.2- fold and the conversion of [^3^H]-pregnenolone into [^3^H]-17α-hydroxyprogesterone was inhibited by 14–17%. These data indicated that 3β-HSD activity (conversion of [^3^H]-pregnenolone to [^3^H]-progesterone) was inhibited.

**Figure 5:**
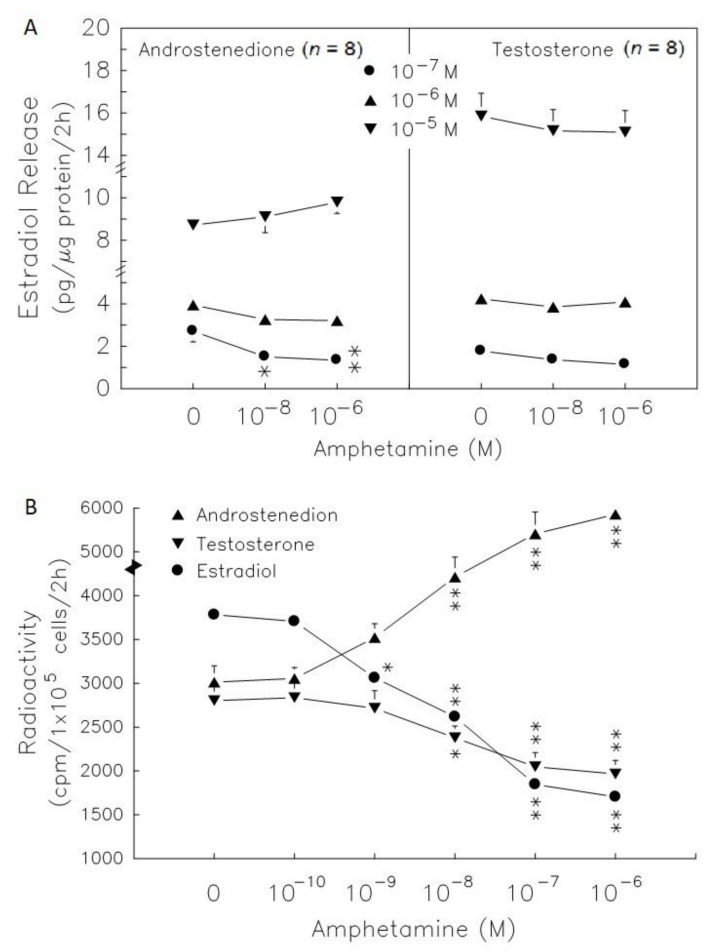
Effect of amphetamine on the activities of 17β-HSD and P450arom in rat granulosa cells. (**A**) The release of estradiol following the presence of androstenedione or testosterone at different doses (○, 0 M; ●, 10^−7^ M; ▲, 10^−6^ M; ▼, 10^−5^ M). (**B**) Rat granulosa cells were incubated with [^3^H]-pregnenolone (10,000 cpm) and different doses of amphetamine at 37 °C for 2 h. The medium was extracted by ether, dried, and then reconstituted in ethanol before analysis by TLC. The radioactivities of [^3^H]-androstenedion (▲), [^3^H]-testosterone (▼), and [^3^H]-estradiol (●) were measured. * *p* < 0.05, ** *p* < 0.01 compared with amphetamine at 0 M, respectively. Each symbol represents mean ± s.e.m.

Androstenedione administration at 10^−^^7^ to 10^−^^5^ M dose-dependently increased estradiol secretion by 2.7-, 3.9- and 8.5-fold (*p* < 0.01, Figure 5A, left panel). Amphetamine at 10^−^^6^ M decreased estradiol release by 59%, and 50% in the presence of 10^−^^8^ M (see lower panel of Figure 1, acting as a historical control) and 10^−^^7^ M androstenedione (*p* < 0.01, Figure 5A). However, amphetamine did not alter estradiol release in the presence of 10^−^^6^ M and 10^−^^5^ M androstenedione. Testosterone administration at 10^−^^7^ to 10^−^^5^ M dose-dependently increased estradiol secretion by 1.7-, 4.2- and 15.5-fold (*p* < 0.05 or *p* < 0.01, Figure 5A, right panel). The estradiol levels released were not altered after treatment with amphetamine (10^−^^6^ M) and testosterone in granulosa cells.

Moreover, 17β-HSD activity (conversion of [^3^H]-androstenedione to [^3^H]-testosterone) also decreased by ~18% when amphetamine at 10^−^^8^ and 10^−^^6^ M was employed (Figure 5B). [^3^H]-estradiol production decreased by 20–55% in the presence of amphetamine at 10^−^^9^ to 10^−^^6^ M (Figure 5A).

### 3.3. Intracellular Calcium Role in the Amphetamine Effect on Progesterone and Estradiol Secretion

**Figure 6:**
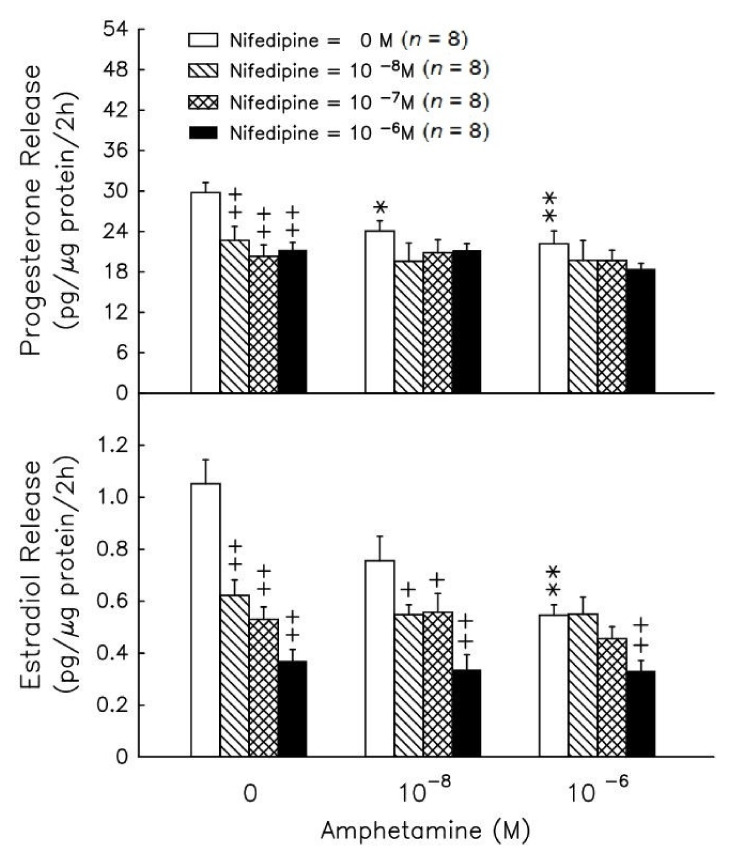
Effect of amphetamine on the release of progesterone (upper panel) and estradiol (lower panel) in rat granulosa cells with graded concentrations of nifedipine. To evaluate estradiol production, androstenedione was added to a final concentration of 10^−8^ M. After incubation for 2 h, media were collected and stored at −20 °C until analyzed for progesterone and estradiol by RIA. * *p* < 0.05, ** *p* < 0.01 compared with amphetamine at 0 M, respectively. ^+^ *p* < 0.05, ^++^ *p* < 0.01 compared with the non-nifedipine-treated group, respectively. Each symbol represents mean ± s.e.m.

Amphetamine at 10^−^^8^–10^−^^6^ M resulted in a significant decrease (*p* < 0.01) in progesterone (Figure 6, upper panel), but amphetamine only exhibited a significant decrease in estradiol release at 10^−^^6^ M (Figure 6, lower panel). The addition of nifedipine (an L-type calcium channel blocker) did not yield further suppressive effects of amphetamine on the release of progesterone (Figure 6, upper panel). However, amphetamine was capable of further suppressing the release of estradiol release under the presence of nifedipine at 10^−^^6^ M (Figure 6, lower panel).

**Figure 7:**
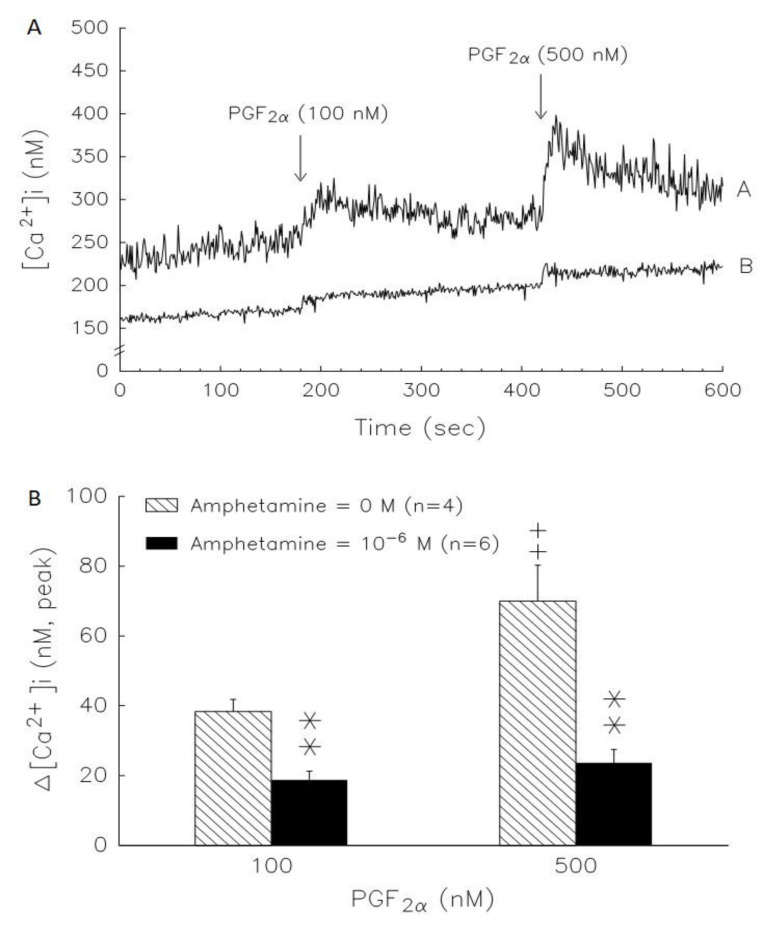
A representative result of the time course of amphetamine effect on basal and PGF2α-stimulated increases of [Ca^2+^]i in rat granulosa cells. (**A**) Cells were loaded with Fura-2/AM for 30 min, washed, and incubated with loading buffer containing 2 mM in the (line A) absence (*n* = 4) and (line B) presence (*n* = 6) of 10^−6^ M amphetamine for 2 h. The addition of PGF2α at final concentrations of 100 nM or 500 nM is indicated by an arrow and the fluorescence of Fura-2 and Fura-2-Ca^2+^ was calculated and the graph was drawn by Sigma Plot. (**B**) Inhibitory effects of amphetamine on PGF2α-induced increase of [Ca^2+^]i in rat granulosa cells. The increase of [Ca^2+^]i induced by PGF2α was calculated as the difference between basal [Ca^2+^]i (before the addition of PGF2α) and the maximal levels of [Ca^2+^]i obtained after the addition of PGF2α. ** *p* < 0.01 compared with amphetamine at 0 M. ^++^ *p* < 0.01 compared with PGF2α at 100 nM, respectively. Each column represents mean ± s.e.m.

We examined the PGF2α effect on [Ca^+^]i in rat granulosa cells (Figure 7A, line A). PGF2α at 100 and 500 nM displayed rapid, transient and dose-dependent [Ca^2+^]i elevation. The initial rapid [Ca^2+^]i phase was followed by a sustained phase that continued for more than 5 min. The data in Figure 7A, line B, show that amphetamine pretreatment was able to significantly (*p* < 0.01) decrease basal [Ca^2+^]i (before PGF2α stimulation) and attenuate PGF2α stimulation on [Ca^2+^]i. Both the rapid and sustained phases elicited by PGF2α were blocked by amphetamine pretreatment (Figure 7A). The increase in [Ca^2+^]i induced by PGF2α was calculated as the difference between the basal [Ca^2+^]i and maximal [Ca^2+^]i levels following PGF2α treatment. Without amphetamine pretreatment, the increases in [Ca^2+^]i induced by 100 nM and 500 nM PGF2α were 38.4 ± 3.5 and 70.0 ± 10.2 nM, respectively. The increase in [Ca^2+^]i induced by PGF2α was significantly diminished by amphetamine pretreatment (*p* < 0.01, Figure 7B).

## 4. Discussion

The major findings from this investigation are (i) that pFSH-induced progesterone and estradiol production were inhibited by amphetamine in rat granulosa cells, whereas amphetamine promoted the pFSH-induced intracellular cAMP levels in granulosa cells; (ii) the addition of 8-Br-cAMP, a cAMP donor, still could not recover the inhibition of progesterone and estradiol production in amphetamine-treated granulosa cells, and there were no further inhibitory effects of combined amphetamine and H89 (i.e., PKA inhibitor); (iii) amphetamine inhibited the activities of PKA-downstream steroidogenic enzymes (i.e., P450scc, 3β-HSD, 17β-HSD and P450arom); (iv) amphetamine inhibited calcium influx-induced progesterone/estradiol production by suppressing L-type calcium channel activity.

The most interesting findings from this investigation are that amphetamine directly inhibits FSH-induced progesterone/estradiol production in a dose-response manner in rat granulosa cells (Figure 1). Here, we found the effective dose of amphetamine in reducing progesterone and estradiol secretion by rat granulosa cells in vitro to be 10^−8^–10^−6^ M (≈3.86–386 ng/mL), which is lower than the effective doses (1–3 mg/kg body weight) that were employed to change behavior in vivo [19,38,39]. Likewise, our selected incubation doses and duration were based on previous human clinical findings by Angrist and colleagues that an acute oral amphetamine administration (0.25–0.5 mg/kg) could markedly raise plasma amphetamine levels to ~2.2–5.2 × 10^−^^7^ M (≈30–70 ng/mL), peaking at 2–3 h [33]. Thus, our present findings further verify the cellular hormonal biosynthetic responses to physiological amphetamine levels in rat granulosa cells. Although amphetamine has been shown to impair female reproductive behaviors, still little is known about the endocrinological role of amphetamine in female reproductive function. To our knowledge, this is the first study demonstrating that female reproductive hormonal production can be perturbed by physiological levels of amphetamine, and the obtained results are similar to previous reports of which amphetamine negatively regulates both basal and hCG-stimulated testosterone release in vivo and in vitro. It has been demonstrated that amphetamine-impaired testosterone production occurs, for instance, as a result of interference with the post-cAMP signaling system, decreased L-type calcium channel activity, and suppressed subsequent steroidogenic enzyme activities. However, there is still a lack of evidence regarding the cellular mechanisms for the perturbations of amphetamine on hormonal production function in rat granulosa cells.

cAMP/PKA signaling plays one of the critical roles in gonadotropin-stimulated sex hormone release, and several previous studies have shown that amphetamine may interfere with sex hormone secretion through the cAMP/PKA pathway. Amphetamine induces an increase in cAMP production but suppresses testosterone production in testicular interstitial cells [9,10]. These existing results suggest that amphetamine inhibition of male sexual hormone production does not directly decrease intracellular cAMP content. Based on the similarity in sex hormone production between genders, amphetamine could impair hormonal biosynthetic responses in females. However, it is not known whether such a mechanism also occurs in the granulosa cells treated with amphetamine. Here, we observed that amphetamine substantially promoted pFSH-induced intracellular cAMP levels in granulosa cells, but that cAMP-induced progesterone and estradiol production were inhibited by amphetamine treatment (Figure 2). To evaluate whether the amphetamine effect is dependent upon cAMP or PKA, we used 8-Br-cAMP, a cAMP donor, to increase intracellular cAMP content and the subsequent production of estradiol and progesterone (Figure 3). Of interest, we found that the 8-Br-cAMP-induced production of progesterone and estradiol was suppressed by amphetamine in granulosa cells (Figure 3). Thus, we demonstrated that amphetamine, at least in part, attenuates sex hormone-release through cAMP-related pathways. On the other hand, we used H89 (i.e., PKA inhibitor) to examine the contribution of the PKA downstream cascade, one of the primary cAMP-mediated modes of signaling, to the inhibitory effects of amphetamine on sex hormone production. We observed that the addition of H89 did not yield further suppressive effects of amphetamine on the release of estradiol and progesterone (Figure 3), suggesting that amphetamine directly inhibits PKA-stimulated sex hormone production. However, we still could not rule out the possibility that there were other existing cAMP-mediated cellular signaling systems involved.

Previous reports demonstrate that PKA activates the ability of its downstream steroidogenic enzymes to synthesize progesterone and estradiol in rat granulosa cells [25,35]. Therefore, we further measured PKA-downstream steroidogenic enzyme activities in amphetamine-reduced progesterone and estradiol production, although we had demonstrated the PKA involvement in amphetamine’s inhibitory effects (Figure 3). Our present study also showed that amphetamine attenuated the stimulatory effect of 25-OH-cholesterol on progesterone release. Pregneolone administration increased progesterone release, and the stimulatory effects were also attenuated by amphetamine (Figure 4A). This suggests that amphetamine attenuated not only the activity of P450scc, the rate-limiting enzyme for progesterone biosynthesis, but also the microsomal enzyme 3β-HSD. Our findings that amphetamine increased [^3^H]-pregnenolone and decreased [^3^H]-progesterone indicate the inhibition of 3β-HSD activity in rat granulosa cells (Figure 4B). On the other hand, the dose-dependent inhibition of estradiol release caused by amphetamine was diminished by androstenedione or testosterone (Figure 5A). Moreover, the increase in [^3^H]-androstenedione and the decreases in [^3^H]-testosterone/[^3^H]-estradiol by amphetamine (Figure 5B) indicated that the activities of both 17β-HSD and P4Acc50arm were inhibited by amphetamine. Taken together, amphetamine might inhibit progesterone/estradiol production by reducing steroidogenic enzyme activities (i.e., P450scc, 3β-HSD, 17β-HSD and P450arom) in rat granulosa cells.

Calcium ions play an important role in steroidogenesis control in granulosa cells [40,41], and L-type Ca^2+^ channels also have been identified using the patch-clamp technique in chicken granulosa cells [42]. The underlying molecular mechanisms are Ca^2+^, calmodulin or Ca^2+^/calmodulin-dependent protein kinases (CaMKs), which play primary roles in the regulation of MAPK activity in various types of cells [43,44,45]. FSH-induced ERK activity in rat granulosa cells is partially mediated by an increase in Ca^2+^ influx, and an increase in [Ca^2+^]i promotes ERK phosphorylation [46]. Moreover, several lines of evidence also indicate that cAMP, PKA, [Ca^2+^]i, CaMK and MAPK can regulate the activity of related transcription factors in steroidogenic cells [47,48,49,50]. It has been suggested that the calcium ion contributes to the amphetamine signal transduction pathway [10,51]. Amphetamine has a biphasic action on Ca^2+^ influx in the neurons of the snail Lymnaea, causing activation at 10^−9^–10^−7^ M and inhibition at higher concentrations [52]. We further evaluated the role of Ca^2+^ in amphetamine-deceased steroidogenesis using a commonly used L-type Ca^2+^ channel blocker, nifedipine [10,53]. Either nifedipine or amphetamine could decrease estradiol and progesterone release in the present study. Amphetamine administration showed an inhibitory effect of nifedipine on estradiol release but not progesterone production (Figure 6), implying that there might be an alternative mechanism for amphetamine’s inhibitory effects on estradiol production, which should be independent of L-type Ca^2+^ channels. Furthermore, we examined whether the increase in [Ca^2+^]i by PGF2α would be affected by amphetamine, and our data showed that amphetamine pretreatment for 2 h decreased both basal and PGF2α-induced [Ca^2+^]i (Figure 7A). Moreover, the maximum increases in [Ca^2+^]i induced by 100 nM and 500 nM PGF2α were significantly diminished by amphetamine pretreatment (Figure 7B). Taken together, amphetamine inhibited calcium influx-induced progesterone and estradiol production by suppressing L-type calcium channel activity in granulosa cells.

To our knowledge, there are no investigations directly examining the effect of amphetamine on sex hormone production in female granulosa cells. However, several reports have revealed that cocaine- and amphetamine-regulated transcript (CART), a neuropeptide protein, is capable of negatively regulating female sex hormone release [53,54,55,56,57]. For example, CART was expressed both in granulosa cells and theca cells [54]. CART signaling has been reported to inhibit the ability of subordinate follicles to synthesize aromatase and produce estradiol [55,56], and is associated with the selection of the dominant follicle [55]. Additionally, CART inhibits FSH-induced granulocyte proliferation and estradiol production in porcine ovarian follicular granulosa cells [54]. More importantly, Sen et al. demonstrated that CART inhibits FSH-induced cAMP accumulation, Ca^2+^ influx, and aromatase mRNA expression [53]. CART has been reported to inhibit the activation of cAMP downstream cascades (e.g., extracellular signal-regulated kinase 1/2 and protein kinase B/Akt), thereby decreasing sex hormone production [57]. Taken together, these findings imply a possible regulatory role of CART proteins in the mechanism of estradiol production inhibited by amphetamine in granulosa cells, but the exact underlying mechanism still requires further investigation.

Although there are many mechanisms involved in estrogen and progesterone production, previous studies have indicated that this hormonal production is mainly regulated through PKA and calcium channel stimulation. Therefore, in our study, we used the inhibitors of these two above pathways to investigate the possible impacts of these relevant cellular signaling pathways. However, we still cannot exclude the involvement of other intracellular signaling mechanisms (e.g., CART proteins, StAR protein, SF-1, ERK), which warrant further investigation and analysis in future studies. In addition, we did not perform toxicological analysis in this study, thus we cannot rule out the possible influence of the toxic response of amphetamine on the above-mentioned estrogen/progesterone production mechanisms. Although the effects of lower doses of amphetamine on hormone secretion were not evaluated in this study, experiments with more sensitive radiation treatments did show that amphetamine at lower doses still had the effect of inhibiting the synthesis of certain steroid hormone enzymes. However, it has to be noted that the amphetamine incubation concentrations used in this study are within the physiological range reported by a previous human clinical study [33]. Additionally, a recovery experiment would be warranted for further study to better clarify whether there are possible toxic effects involved. In this study, our cell culture experimental method was mainly based on the incubation time (two hours) used in previous studies [25,34], thus we could not confirm whether this incubation time or low dose achieved the biological effect of amphetamine stimulation. Based on the results of the present study, although we confirmed that amphetamine interferes with progesterone and estradiol production, the basis for these obtained results is cellular approaches. Future in vivo studies and human studies are warranted for further applications in human populations.

## 5. Conclusions

**Figure 8:**
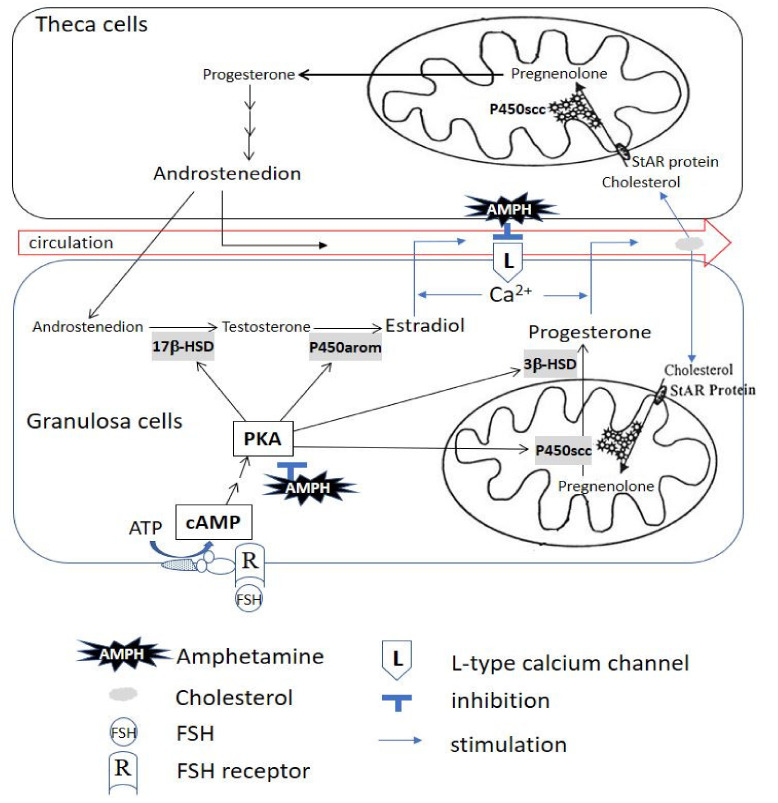
The illustrated scheme demonstrates that amphetamine inhibits progesterone and estradiol secretion by suppressing PKA-downstream steroidogenic enzyme activity and L-type calcium channel-mediated Ca^2+^ influx in rat granulosa cells. The primary involved PKA-downstream molecules are P450scc, 3β-HSD, 17β-HSD and P450arom. AMPH: amphetamine; ATP: adenosine triphosphate; Ca^2+^: calcium; cAMP: adenosine 3′:5′-cyclic monophosphate; FSH: follicle-stimulating hormone; L: L-type calcium channel; PKA: protein kinase A; P450arom: cytochrome P450 aromatase; P450scc: cytochrome P450 side-chain cleavage; R: FSH receptor; StAR Protein: steroidogenic acute regulatory protein; 3β-HSD: 3β-hydroxysteroid dehydrogenase; 17β-HSD: 17β-hydroxysteroid dehydrogenase.

In summary, we demonstrated that amphetamine inhibits progesterone and estradiol secretion by suppressing PKA-downstream steroidogenic enzyme activity (i.e., P450scc, 3β-HSD, 17β-HSD and P450arom) and L-type calcium channels in rat granulosa cells. Our current findings suggest the possible involved mechanism(s) for amphetamine affecting female sex hormone production perturbations at cellular level. A diagram of the general scheme for the possible effects of amphetamine on progesterone/estradiol secretion in rat granulosa cells is presented in Figure 8.

## Data Availability

The data presented in this study are available upon request from the corresponding authors.
